# Validation of ablation site classification accuracy and trends in the prediction of potential reconnection sites for atrial fibrillation using the CARTONET® R12.1 model

**DOI:** 10.1002/joa3.13131

**Published:** 2024-08-13

**Authors:** Wataru Sasaki, Naomichi Tanaka, Kazuhisa Matsumoto, Daisuke Kawano, Masataka Narita, Tsukasa Naganuma, Kenta Tsutsui, Hitoshi Mori, Yoshifumi Ikeda, Takahide Arai, Kazuo Matsumoto, Ritsushi Kato

**Affiliations:** ^1^ Department of Cardiology Saitama Medical University, International Medical Center Hidaka Saitama Japan; ^2^ Department of Cardiology Higashimatsuyama Medical Association Hospital Higashimatsuyama Saitama Japan

**Keywords:** automatic classification, CARTONET®, external validation, machine learning, pulmonary vein isolation

## Abstract

**Background:**

CARTONET® enables automatic ablation site classification and reconnection site prediction using machine learning. However, the accuracy of the site classification model and trends of the site prediction model for potential reconnection sites are uncertain.

**Methods:**

We studied a total of 396 cases. About 313 patients underwent pulmonary vein isolation (PVI), including a cavotricuspid isthmus (CTI) ablation (PVI group) and 83 underwent PVI and additional ablation (i.e., box isolation) (PVI+ group). We investigated the sensitivity and positive predictive value (PPV) for automatic site classification in the total cohort and compared these metrics for PV lesions versus non‐PV lesions. The distribution of potential reconnection sites and confidence level for each site was also investigated.

**Results:**

A total of 29,422 points were analyzed (PV lesions [*n* = 22 418], non‐PV lesions [*n* = 7004]). The sensitivity and PPV of the total cohort were 71.4% and 84.6%, respectively. The sensitivity and PPV of PV lesions were significantly higher than those of non‐PV lesions (PV lesions vs. non‐PV lesions, %; sensitivity, 75.3 vs. 67.5, *p* < .05; PPV, 91.2 vs. 67.9, *p* < .05). CTI and superior vena cava could not be recognized or analyzed. In the potential reconnection prediction model, the incidence of potential reconnections was highest in the posterior, while the confidence was the highest in the roof.

**Conclusion:**

The automatic site classification of the CARTONET®R12.1 model demonstrates relatively high accuracy in pulmonary veins excluding the carina. The prediction of potential reconnection sites feature tends to anticipate areas with poor catheter stability as reconnection sites.

## Graphical abstract

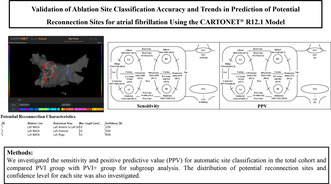

## 1 INTRODUCTION

Machine learning technology has been widely used in the medical field and there are a few reports of the clinical use of machine learning technology in the field of cardiac electrophysiology.^1^, ^2^, ^3^ There have been a few reports on its application in the catheter ablation sector. In the widely used CARTO® 3 system for catheter ablation,^4^ a cloud‐based management system known as CARTONET® (Biosense Webster, Inc., Irvine, CA) has recently become available.^5^, ^6^, ^7^ The CARTONET® R12.1 model allows for the automatic classification of ablation sites in atrial fibrillation (AF) ablation based on machine learning models, as well as a prediction model for potential reconnection sites based on the ablation information at each site. However, external validation of these machine learning‐based site classifications and a detailed examination of the prediction model for the potential reconnection sites have not yet been conducted. The purpose of this study was to investigate the external validation of its site classification model and the characteristics of its prediction model for potential reconnection sites.

## 2 METHODS

### 2.1 Study patients

The study involved 396 patients who underwent a first session of catheter ablation of AF with the CARTO® 3 system (Biosense Webster, Inc., Irvine, CA) at a single facility between March 30, 2020, and July 10, 2023. Cases with nonpulmonary vein (PV) foci originating from areas other than the left atrial posterior wall and the superior vena cava (SVC) were excluded from this analysis. We divided the cases into two groups: the pulmonary vein isolation (PVI) group, in which the ablation procedure consisted of a PVI and cavo‐tricuspid isthmus (CTI) ablation and the PVI+ group, in which the ablation procedure consisted of not only a PVI and CTI but also a Box isolation or SVC isolation.

This study was performed in accordance with the provisions of the Declaration of Helsinki and local regulations. The research protocol was approved by the Hospital's Institutional Review Board (2023‐070).

### 2.2 Ablation procedure

The ablation procedure was performed under general anesthesia with esophageal temperature sensor in all cases. All patients underwent ablation with the THERMOCOOL SMARTTOUCH®SF catheter (STSF; Biosense Webster, Inc., Irvine, CA) or Q DOT‐MICRO™ catheter (QDOT; Biosense Webster, Inc., Irvine, CA). The ablation protocol was as follows; with the case of using STSF, In PVI, ablation was performed with a contact force of 10–20 g and a setting wattage of 35–40 W, targeting an Ablation Index (AI) of 450–500. For lesions near the esophagus, ablation was conducted at 40 W for 7 s or until the esophageal temperature reached 39°C. The CTI protocol was similar to PVI. SVC isolation employed the same contact force and setting wattage as PVI, targeting an AI of 350.^8^ With the case of using Q DOT, In PVI, conventional‐power temperature‐controlled mode (setting wattage is 50 W, target temperature 47°C, target an AI of 450; ablation stopped automatically if temperature increased above 55°C) and Q mode+, using a very high power, short duration workflow (90 W, 4 s target temperature 55°C; ablation stopped automatically if temperature increased above 60°C) were used in combination. Ablation near the esophagus was exclusively performed using Q mode+. For CTI, a setting wattage of 35 W was used, targeting an AI of 450 or ablation continued until reaching a duration of 30 s. SVC isolation was performed using Q mode+. All ablation procedure was performed using a point‐by‐point technique.

### 2.3 Automatic classification of ablation sites and the potential reconnection prediction with the CARTONET® function

**FIGURE 1:**
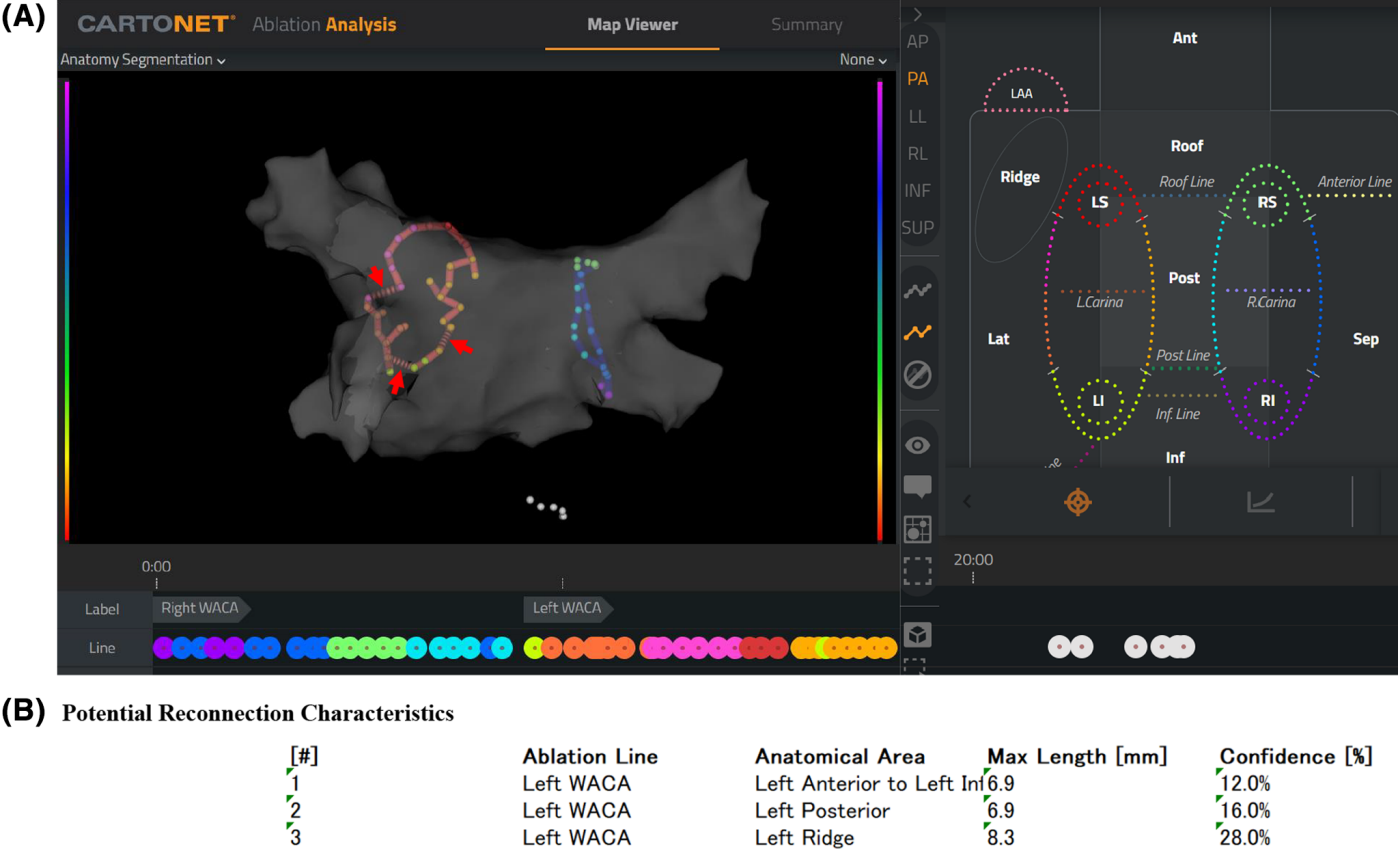
Automatic classification of the ablation sites and potential reconnection prediction. (A) Each ablation site is identified with a color tag, based on the machine learning model (left panel). They are classified into 5 segments (right roof, right anterior, right inferior, right posterior, and right carina) for the right pulmonary veins, 6 segments (left roof, ridge, left anterior, left inferior, left posterior, left carina) for the left pulmonary veins, and 5 segments (roof line, post line, inferior line, mitral line, anterior line) for the sites of the line ablation outside the pulmonary veins (Figure 1A, right panel) with each color tag. The white tag represents the unidentifiable sites. The red arrow represents the dotted line of the potential reconnection sites. (B) The confidence of each potential reconnection site was calculated from the machine learning algorithm.

Figure 1A shows a representative case using CARTONET®. With CARTONET®, the ablation lines are classified into 5 segments (right roof, right anterior, right inferior, right posterior, and right carina) for the right PVs (RPV) and 6 segments (left roof, ridge, left anterior, left inferior, left posterior, and left carina) for the left PVs (LPVs) (Figure 1A, right panel). Line ablation outside the PVs is also classified into 5 segments (roof line, post line, inferior line, mitral line, and anterior line) based on the ablation sites. Based on the machine learning model, these anatomical locations are automatically classified, and each area is color‐coded for display. Points that are automatically classified are connected by lines, and locations predicted by the machine learning model as potential reconnection sites are indicated with dotted lines (Figure 1A, red arrow). These are shown with the distance between the two points and the potential for reconnections being displayed as a percentage (Figure 1B).

### 2.4 External validation of the automatic site classification model

**FIGURE 2:**
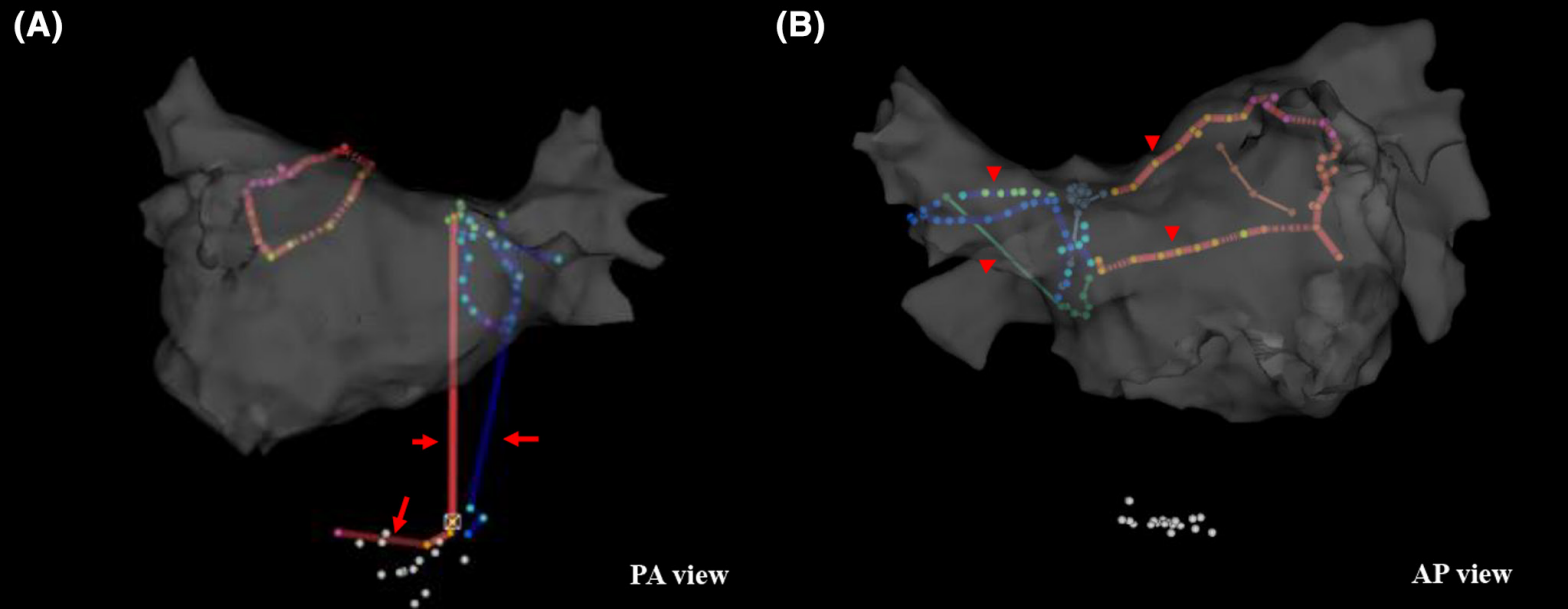
Examples of an inaccurate classification of the ablation lines. The representative cases of an inaccurate classification of the ablation lines. The ablation sites at the cavotricuspid isthmus were wrongly annotated for the PV ablation line (A, red arrow). The ablation sites at the superior vena cava were wrongly annotated for the right PV isolation line, and the sites at the roof and bottom were wrongly annotated for the left PV isolation line (B, red arrowhead).

In the site classification model, the ablation sites were sometimes classified inaccurately (Figure 2). To examine the validity of the learning model, a comparison was performed between the anatomical accuracy of each point automatically classified by machine learning and the anatomical positions as identified by electrophysiologists. The analysis of the ablation lines was conducted by a total of eight electrophysiologists. For each case, a double‐check was performed by two electrophysiologists and in cases where their opinions differed, a discussion was conducted to determine the location. A total of 29 422 points were validated. For each point, a comparison was made between the location classified by the machine learning model and its actual location, and from these results, an examination was conducted regarding the sensitivity and positive predictive value of the machine learning model. For each site, the sensitivity and positive predictive value were calculated using the following formula: Sensitivity = ([Number of points annotated by CARTONET®] − [Number of incorrect points annotated by CARTONET®])/(Number of points annotated by electrophysiologists): Positive predictive value (PPV) = ([Number of points annotated by CARTONET®] − [Number of incorrect points annotated by CARTONET®])/(Number of points annotated by CARTONET®).

We compared the sensitivity and PPV between the PV lesions and non‐PV lesions. We also compared these metrics between the PVI group and the PVI+ group.

### 2.5 Characteristics of potential reconnections prediction model

Using the potential reconnection prediction model, the number of potential reconnection sites was evaluated. The distribution of the potential reconnection sites and the confidence level for each site were also investigated.

### 2.6 Statistical analysis

The statistical analyses were performed using JMP® Pro software, version 16.0 (SAS Institute, Cary, NC, USA). Data are expressed as the mean ± SD for the parametric data and as the median with the IQR for the nonparametric data. The continuous variables were compared using a *t*‐test for the parametric data and Mann–Whitney test for the nonparametric data. The categorical data were compared by a chi‐square test. Two‐sided *p*‐values <.05 were considered statistically significant.

## 3 RESULTS

### 3.1 Clinical characteristics

Table 1 shows the baseline characteristics of study populations. In the PVI+ group, where a BOX isolation or SVC isolation was performed, the proportion of paroxysmal atrial fibrillation was lower (27 [7%] vs. 153 [39%], *p* = .008), and the frequency of male patients was significantly lower (49 [41%] vs. 242 [77.3%], *p* = .0008). Regarding the height, there was a tendency for the individuals in the PVI group to be taller (165.2 ± 8.9 vs. 162.5 ± 9.9, *p* = .02).

**TABLE 1:** Patient characteristics.

	All (*n* = 396)	PVI group (*n* = 313)	PVI+ group (*n* = 83)	*p* value
Age (years)	69 ± 10	69 ± 10	70 ± 11	.77
Height (cm)	164.6 ± 9.2	165.2 ± 0.5	162.5 ± 1.0	<.05*
Body weight (kg)	67.6 ± 32.2	68.9 ± 35.4	62.7 ± 14.4	.06
Gender, male, *n* (%)	291 (73.5%)	242 (77.3%)	49 (41.0%)	<.05*
CHAD2 score	1.2 ± 1.0	1.2 ± 1.0	1.3 ± 1.1	.80
LVEF (%)	56.9 ± 16	57.2 ± 16	56 ± 16	.72
Paroxysmal AF, *n* (%)	180 (46%)	153 (39%)	27 (7%)	<.05*
LA diameter, mm	43.3 ± 6.8	43 ± 6.6	44 ± 7.5	.07

*Note*: The continuous variables are shown as the mean ± SD and the categorical variables as the number (%).

Abbreviations: AF, atrial fibrillation; LA, left atrium; LVEF, left ventricular ejection fraction.

* *p* < .05.

### 3.2 Validation of the automatic site classification model

**FIGURE 3:**
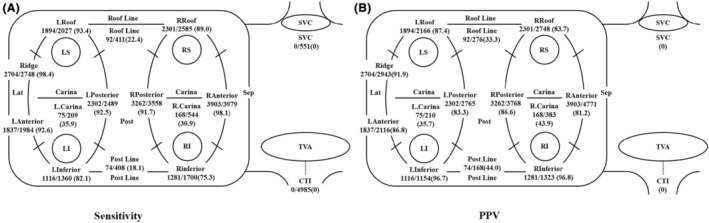
Sensitivity and positive predictive values of the automatic classification of the ablation sites. (A) The sensitivity calculated on the automatic classification of the ablation sites, including all points. (B) The positive predictive value calculated on the automatic classification of the ablation sites, including all points.

A total of 29,422 points were analyzed (PVI group [*n* = 22 418], PVI+ group [*n* = 7004]). The sensitivity and PPV of the total cohort were 71.4% and 84.6%, respectively. The sensitivity and PPV of PV lesions were significantly higher than those of non‐PV lesions (PV lesions vs. non‐PV lesions, %; sensitivity, 75.3 vs. 67.5, *p* < .05; PPV, 91.2 vs. 67.9, *p* < .05). Figure 3 shows the sensitivity and PPV of automatic site classification for each segmentation, calculated based on the total points at each segmentation.

Figure 3A shows the sensitivity. For the right PVs, the sensitivity was as follows: R inferior, 1281/1700 (75.3%); R anterior, 3903/3979 (98.1%); R roof, 2301/2585 (89.0%); R posterior, 3262/3558 (91.7%); R Carina, 168/554 (30.9%). For the left PVs, the sensitivity was as follows: L inferior, 1116/1360 (82.1%); L anterior, 1837/1984 (92.6%); Ridge, 2704/2748 (98.4%); L roof, 1894/2027 (93.4%); L posterior, 2302/2489 (92.5%); L Carina, 75/209 (35.9%). For the roofline, the sensitivity was 92/411 (22.4%). For the post line, the sensitivity was 74/408 (18.1%).

Figure 3B shows the PPV. For the right PVs, the PPV was as follows: R inferior, 1281/1323 (96.8%); R anterior, 3903/4771 (81.2%); R roof, 2301/2748 (83.7%); R posterior, 3262/3768 (86.6%); R Carina, 163/383 (43.9%).

For the left PVs, the PPV was as follows: L inferior, 1116/1154 (96.7%); L anterior, 1837/2116 (86.8%); Ridge, 2704/2943 (91.9%); L roof, 1894/2166 (87.4%); L posterior, 2302/2765 (83.3%); L Carina, 75/210 (35.7%). For the roofline, the PPV was 92/276 (33.3%). For the post line, the PPV was 74/168 (44.0%). The CTI and SVC were not recognized or analyzed by the R12.1 model.

**FIGURE 4:**
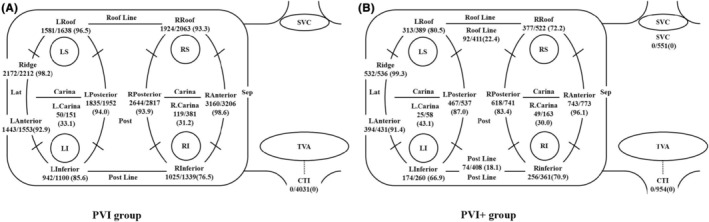
Sensitivity of the automatic classification of the ablatio sites (A, PVI group, B, PVI+ group).

**FIGURE 5:**
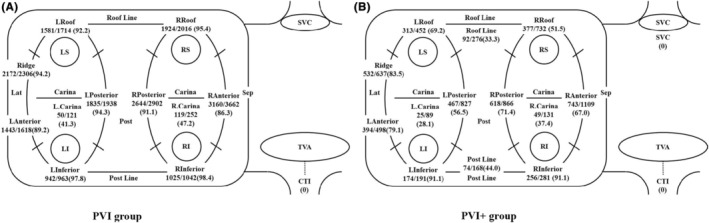
Positive predictive values of the automatic classification of the ablation sites (A; PVI group, B; PVI+ group).

Figures 4 and 5 show a comparison of sensitivity and PPV between the PVI group and PVI+ group.

For the right PVs, the sensitivity was as follows: PVI group (Figure 4A) versus PVI+ group (Figure 4B); R inferior, 1025/1339 (76.5%) versus 256/361 (70.9%), *p* = .273; R anterior, 3160/3206 (98.6%) versus 743/773 (96.1%), *p* = .537; R roof, 1924/2063 (93.3%) versus 377/522 (72.2%), *p* < .05; R posterior, 2644/2817 (93.9%) versus 618/741 (83.4%), *p* < .05; R Carina, 119/381 (31.2%) versus 49/163 (30.0%), *p* = .821. For the left PVs, the sensitivity was as follows: PVI group versus PVI+ group; L inferior, 942/1100 (85.6%) versus 174/260 (66.9%), *p* < .05; L anterior, 1443/1553 (92.9%) versus 394/431 (91.4%), *p* = .774; Ridge, 2172/2212 (98.2%) versus 532/536 (99.3%), *p* = .078; L roof, 1581/1638 (96.5%) versus 313/389 (80.5%), *p* < .05; L posterior, 1835/1952 (94.0%) versus 467/537 (87.0%), *p* = .642; L Carina, 50/151 (33.1%) versus 25/58 (43.1%), *p* = .280. The sensitivity was significantly higher for the R roof, R posterior, L inferior, and L roof in the PVI group.

For the right PVs, the PPV was as follows: PVI group (Figure 5A) versus PVI+ group (Figure 5B); R inferior, 1025/1042 (98.4%) versus 256/281 (91.1%), *p* = .272; R anterior, 3160/3662 (86.3%) versus 743/1109 (67.0%), *p* < .05; R roof, 1924/2016 (95.4%) versus 377/732 (51.5%), *p* < .05; R posterior, 2644/2902 (91.1.%) versus 618/866 (71.4%), *p* < .05; R Carina, 119/252 (47.2%) versus 49/131 (37.4%), *p* = .169. For the left PVs, the PPV was as follows: PVI group versus PVI+ group; L inferior, 942/963 (97.8%) versus 174/191 (91.1%), *p* = .388; L anterior, 1443/1618 (89.2%) versus 394/498 (79.1%), *p* < .05; Ridge, 2172/2306 (94.2%) versus 532/637 (83.5%), *p* < .05; L roof, 1581/1714 (92.2%) versus 313/452 (69.2%), *p* < .05; L posterior, 1835/1938 (94.3%) versus 467/827 (56.5%), *p* < .05; L Carina, 50/121 (41.3%) versus 25/89 (28.1%), *p* = .112. The PPV was significantly higher for the R anterior, R roof, R posterior, L anterior, Ridge, L roof, and L posterior regions in the PVI group.

### 3.3 The frequency of potential reconnection sites and the percentage of potential reconnections

Table 2 shows the results of the potential reconnection site analysis. A total of 711 segments were predicted as potential reconnection sites. The incidence of potential reconnection sites of the left pulmonary veins (LPVs) (*n* = 444 [62.4%]) was higher than that of the right pulmonary veins (RPVs) (*n* = 267 [37.6%]) (Table 2 left panel). Among each PV, the right and left posterior areas had the highest incidence of potential reconnection sites. The confidence of the potential reconnection sites was highest in the roof area for both the RPVs and LPVs (RPVs, 14% [4–44%]; LPVs, 16% [8–21%]) (Table 2 right panel).

**TABLE 2:** Characteristics of the potential reconnection prediction model.

Distribution of the potential reconnection sites	
Right WACA: 267/711 (37.6%)	Left WACA: 444/711 (62.4%)
Right Anterior: 84/267 (31.5%)	Left Anterior: 55/444 (12.4%)
Right Inferior: 46/267 (17.2%)	Left Inferior: 70/444 (15.8%)
Right Posterior: 101/267 (37.8%)	Left Posterior: 183/444 (41.2%)
Right Roof: 36/267 (13.5%)	Left Ridge: 91/444 (20.5%)
	Left Roof: 45/444 (10.2%)

Confidence of the potential reconnection sites	
Right WACA	Left WACA
Right Anterior: 8 [4–22] %	Left Anterior: 10 [6–18] %
Right Inferior: 8 [4.5–24] %	Left Inferior: 10 [6–16.5] %
Right Posterior: 8 [4–18] %	Left Posterior: 10 [6–18] %
Right Roof: 14 [4–44] %	Left Ridge: 12 [8–16] %
	Left Roof: 16 [8–21] %

*Note*: The left panel shows the distribution of the potential reconnection sites. The incidence of potential reconnection sites for the LPVs was higher than that for the RPVs. Among each PV, the right and left posterior areas had the highest incidence of potential reconnection sites. The right panel shows the confidence of the potential reconnection sites. The confidence of the potential reconnection sites was highest for the roof area for both the RPVs and LPVs.

Abbreviations: LPV, left pulmonary vein; PV, pulmonary vein; RPV, right pulmonary vein; WACA, wide area circumferential ablation.

## 4 DISCUSSION

### 4.1 Major findings

The major findings of our study were as follows:

(1) The CARTONET® R12.1 model allows for a relatively high accuracy of the automatic classification of each part of the PV lesions. (2) Compared to the cases in the PVI+ group, the sensitivity and PPV in the PVI group were higher for most segments. (3) The accuracy of the automatic classification outside the PVI line was relatively low, and it was unable to identify the SVC and CTI lines. (4) In the potential reconnections prediction model, the incidence of potential reconnections was highest for the posterior area, while the confidence was highest for the roof area.

#### 4.1.1 Accuracy of the automatic site classification model

The automated classification model allows for a relatively high accuracy in most segments. However, the PPV and sensitivity in the PVI+ group were lower in most segments than that in the PVI group. In cases in which a box isolation or SVC isolation was performed, it was difficult to automatically recognize the segments near the ablation lines because of their proximity to the PV line, thereby reducing the accuracy of the automatic site classification. Future improvements in the accuracy may be possible by including more cases with ablation lines near each other in the training model.

#### 4.1.2 The confidence of the potential reconnection prediction model

Regarding the potential reconnection prediction model, the R12.1 model tended to estimate that potential reconnections were more likely to be estimated on the posterior wall of both the left and right PVs. The reason for the higher incidence of potential reconnections in the left PVs compared to the right PVs was because of the esophagus more commonly running along the left side of the left atrium. Esophageal injury resulting from catheter ablation can lead to lethal complications.^9^ To avoid esophageal injury, the catheter is moved in a stepping manner rather than continuously,^10^ and the ablation time is relatively shorter than in other areas, leading to a higher incidence of potential reconnections. As for the right PVs, the vertebrae lie along the ablation line, making it difficult to maintain catheter stability at the same position, causing a higher incidence of potential reconnections.

The confidence in potential reconnections was highest for the roof area of both PVs. That was thought to be because the roof area is the part where the catheter moves the most because of the patient's breathing, making it difficult to obtain good catheter stability as compared to other areas.

### 4.2 Clinical implications

Numerous clinical studies on 3D mapping have been reported thus far, focusing on comparisons of the detailed ablation information of various sites, as well as comparisons of the anatomical positional relationships.^8^, ^11^ In clinical studies using 3D mapping, considerable time was previously required to manually acquire data on the anatomical positions and ablation information for each ablation site. Utilizing CARTONET® enables the automatic classification of AF ablation lines, suggesting a potential reduction in the time needed for research. However, in the R12.1 model for LA segmentation, the accuracy of the automatic recognition for areas near the carina or near a box isolation/SVC isolation is low, necessitating final confirmation by an EP doctor. This model employs a machine learning approach, and it is possible that there may be insufficient training for the SVC isolation lines and posterior wall lines. However, as more cases are learned through deep learning models or similar techniques in the future, there is the potential for an improved accuracy.

A recurrent PVI can lead to future recurrences of AF,^12^, ^13^, ^14^ making the prediction and prevention of reconnections crucial for maintaining sinus rhythm. By using machine learning models, it is possible to predict potential reconnection sites, and if a real‐time analysis becomes feasible, this could contribute to improved procedural outcomes.

### 4.3 Limitations

Our study had several limitations. Firstly, the CARTONET® R12.1 model, being designed for retrospective analysis, does not support a real‐time analysis during procedures, leaving its utility in treatment unclear. Secondly, this model employs a machine learning model based on random forests, and its accuracy may vary when trained with different methodologies such as deep learning. Finally, the potential reconnection prediction model has not been externally validated, and future trials will be needed to validate this model in a larger cohort.

## 5 CONCLUSION

Using a machine learning model enabled the automatic classification of the anatomical positions of the pulmonary veins. However, there was a tendency for a decreased accuracy in cases with a nearby ablation, such as around the SVC or the posterior wall isolation lines. The prediction of potential reconnection sites feature tends to anticipate areas with poor catheter stability as reconnection sites.

## AUTHOR CONTRIBUTIONS

HM and RK provided the study concept and design; HM and WS drafted the manuscript; WS, DK, NT, TN, MN, KM, and KT contributed to the data analysis and interpretation. YI, HA, and KM reviewed and revised the manuscript draft.

## FUNDING INFORMATION

No funding was required for this study.

## CONFLICT OF INTEREST STATEMENT

HM received lecture fees from Biosense Webster Japan and Boston Scientific Japan. Our department received grant support from Boston Scientific Japan and Abbott Medical Japan.

## ETHICAL APPROVAL

The study protocol was approved by the Hospital's Institutional Review Board (IRB number; 2023‐070).

## CODE AVAILABILITY

Available upon request.

## Data Availability

Available upon request.
